# Hexokinase 2 as oncotarget

**DOI:** 10.18632/oncotarget.1563

**Published:** 2013-10-31

**Authors:** Krushna C. Patra, Nissim Hay

**Affiliations:** Massachusetts General Hospital Cancer Center, Harvard Medical School, Boston, MA 02114, USA; Department of Biochemistry and Molecular Genetics, College of Medicine, University of Illinois at Chicago, Chicago, IL, USA and Research & Development Section, Jesse Brown VA Medical Center, Chicago, IL, USA

It is well established that cancer cells differ from most somatic adult cells by their high rate of aerobic glycolysis despite oxidative phosphorylation. This distinct property of cancer cells is often used for tumor imaging in vivo following the administration of the labeled glucose analog, fluoro deoxy glucose (FDG). The high uptake of the labeled FDG in cancer cells can then be detected by positron emission tomography (PET). The elevated glucose metabolism in cancer cells is not necessarily required for the generation of ATP but rather to provide building blocks for nucleic acid, fatty acid, and protein synthesis in the rapidly dividing cancer cells [1]. Thus, if the high rate of glucose metabolism in cancer cells is being used to distinguish cancer cells from normal cells, it could also be exploited to selectively target cancer cells in cancer therapy. However, a major challenge in targeting glucose metabolism for cancer therapy is to identify a strategy that would selectively target cancer cells while sparing normal cells, and that would not dramatically impair systemic metabolic homeostasis. Another posed question is; which enzymatic activities should be chosen as targets for selective cancer therapy? Although several enzymes in glycolysis and in the pathways branching from the canonical glycolysis pathway were found to be elevated in cancer cells, these enzymes are also expressed in the normal cells.

In principle it would be best to target the most committed and irreversible reactions in glucose metabolism. There are three irreversible reactions in glycolysis. The first reaction is catalyzed by hexokinase and the second and third reactions are catalyzed by phosphofructokinase and pyruvate kinase respectively. Ideally it would be ideal if cancer cells could be targeted by cutting down glucose flux at the earliest step in glucose metabolism. The first committed step in glucose metabolism is the ATP dependent conversion of glucose to glucose-6-phosphate (G6P), which is catalyzed by hexokinases (HKs). This step determines the direction and magnitude of glucose flux inside the cells, because G6P is at the branching point that supports not only glycolysis but also the pentose phosphate pathway (PPP), glycogenesis and the hexosamine pathways [2].

In mammals, there are four hexokinase isoforms, HK1, HK2, HK3, and HK4 (also known as glucokinase), encoded by separate genes. While HK1, HK2, and HK3 have high affinity for glucose, HK4 has low affinity for glucose and its expression is restricted to the pancreas and liver. HK3 is usually expressed at low levels and is inhibited in physiological concentrations of glucose. HK1 and HK2 are unique with their ability to bind the outer mitochondrial membrane. They are structurally and functionally similar to each other and both are inhibited by their catalytic product G6P. Both HK1 and HK2 are highly expressed in embryonic tissues. However, while HK1 is ubiquitously expressed in the majority of adult tissues, HK2 is expressed at relatively high level only in adipose tissues, skeletal muscles, and heart [3,5]. Despite its absence or low expression in the majority of adult normal cells, HK2 is expressed at high levels in many cancer cells. Perhaps a positive FDG-PET scan of tumors in vivo is an indirect indication of high HK2 expression in these tumor cells [4] because glucose (or FDG) entry through glucose transporters can be reversible and its intracellular retention is dependent on its phosphorylation.

In our recent published studies [5], we showed that HK2 expression is dramatically elevated in tumors derived from mouse models of lung and breast cancer. By contrast HK2 expression in normal lung and mammary gland is undetectable. Thus, HK2 expression distinguishes cancer cells from normal cells. Importantly the high level of HK2 expression is required for oncogenic transformation in vitro and for tumor initiation in mouse model of non-small cell lung cancer (NSCLC) and breast cancer. In complementary experiments we found that HK2 is required for the tumorigenicity of human NSCLC and breast cancer cells in vitro and in vivo. HK2 is required for tumorigenicity despite the continuous expression of HK1. However, the feasibility of targeting HK2 for cancer therapy remains questionable especially since the germ line deletion of *Hk2* in the mouse causes embryonic lethality. Surprisingly, systemic deletion of *Hk2* in the adult mouse did not elicit any visible overt phenotype and did not impair normal glucose homeostasis, at least under resting conditions. Moreover the systemic deletion of *Hk2* impairs tumor progression in a mouse model of NSCLC. These results provided a genetic proof of concept that HK2 could be a selective therapeutic target for cancer.

**Figure 1:**
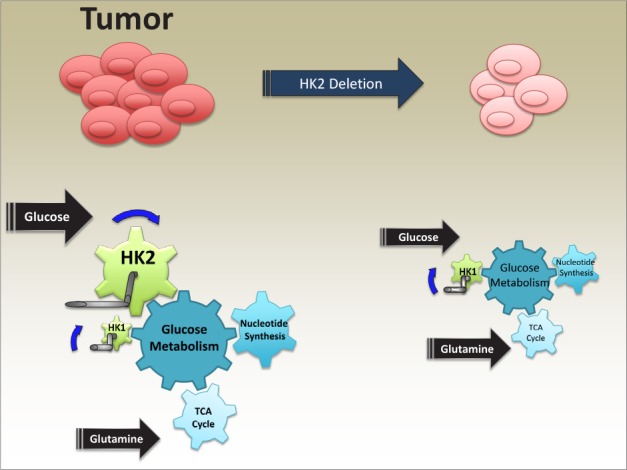
A cartoon depicting HK2 is required for glucose and glutamine metabolism in tumor cells The metabolic pathways inside the cells are like cogwheels inside a machine, controlled by a propelling wheel. In cancer cells, HK2 is a major propelling wheel in glucose metabolism to support accelerated ribonucleotide synthesis and glutamine utilization in the TCA cycle, and to fulfill anabolic demands. HK2 ablation decelerates these pathways resulting in an attenuated tumor growth.

In oncogenic KRas-induced NSCLC cells, HK2 is required, in addition to HK1, for nucleotides biosynthesis via the non-oxidative pentose phosphate pathway, which is a major route of ribonucleotides synthesis in these cells. Indeed, the deletion of HK2 reduced the incorporation glucose-derived deoxyribonucleotides, into DNA synthesis (Fig.1). In addition, HK2 is required for the flow of citrate, derived from glucose, into the TCA cycle, and surprisingly for glutamine utilization in the TCA cycle (Fig1.). The metabolomics studies also suggest that in the absence of HK2 there is a decrease in the serine biosynthesis pathway and fatty acids synthesis. We concluded that while HK1 expression is sufficient for the metabolic demand of normal cells, it could not fulfill the metabolic demand of proliferating cancer cells, which requires the expression of HK2.

Although targeting HK2 for cancer intervention looks promising, the biggest challenge ahead would be to design an isoform specific inhibitor, because of the overlapping enzymatic properties of HK2 with the ubiquitously expressed isoform, HK1. However although HK1 and HK2 are similar to each other there are some differences that could be exploited for the selective inhibition of HK2. For instance, although both isoforms are subjected to feedback inhibition by G6P, inorganic phosphates negate the inhibition of HK1, while sensitizing HK2 inhibition (3). Since the highly metabolic cancer cells usually have higher levels of intracellular inorganic phosphate, a G6P mimetic could preferentially inhibit HK2 in cancer cells.
